# Clinical Reasoning Behind Non-Pharmacological Interventions for the Management of Headaches: A Narrative Literature Review

**DOI:** 10.3390/ijerph17114126

**Published:** 2020-06-09

**Authors:** César Fernández-de-las-Peñas, Lidiane L. Florencio, Gustavo Plaza-Manzano, José L. Arias-Buría

**Affiliations:** 1 Department of Physical Therapy, Occupational Therapy, Physical Medicine and Rehabilitation, Universidad Rey Juan Carlos, 28922 Alcorcón, Spain; lidiane.florencio@urjc.es (L.L.F.); joseluis.arias@urjc.es (J.L.A.-B.); 2 Cátedra Institucional en Docencia, Clínica e Investigación en Fisioterapia, Terapia Manual, Punción Seca y Ejercicio Terapéutico, Universidad Rey Juan Carlos, Alcorcón, 28922 Madrid, Spain; 3 Radiology, Rehabilitation and Physiotherapy Department, Universidad Complutense de Madrid, 28040 Madrid, Spain; gusplaza@ucm.es; 4 Instituto de Investigación Sanitaria del Hospital Clínico San Carlos, 28040 Madrid, Spain

**Keywords:** physical therapy, manual therapy, spinal manipulation, soft tissue, needle, exercise, cognitive, tension type headache, migraine, cervicogenic headache

## Abstract

Headache is the clinical syndrome most commonly observed by neurologists in daily practice. Pharmacological and non-pharmacological treatments are commonly used for the management of headaches; however, the clinical reasoning behind these interventions is not properly applied. We conducted a narrative literature review using as data sources for academic PubMed, MEDLINE, EMBASE, AMED, CINAHL, EBSCO, PEDro, Cochrane Database of Systematic Reviews, Cochrane Collaboration Trials Register, and SCOPUS. This narrative literature review mainly considered systematic reviews, meta-analyses, randomised clinical trials, and expert opinions published after the year 2000 discussing clinical reasoning for application of non-pharmacological interventions in individuals with tension-type, migraine, and cervicogenic headaches. After the data extraction, we organized the literature thematically as follows: (1) mapping of theoretical aspects of non-pharmacological interventions; (2) summarizing most updated literature about effectiveness of non-pharmacological interventions grouped by targeted tissue and headache; (3) identifying research gaps in the existing literature and proposing hypotheses for better understanding of current clinical reasoning. We found that there are many non-pharmacological treatment strategies used for headaches, including beyond the tissue-based impairment treatments (bottom-up) and strategies targeting the central nervous system (top down). Bottom-up strategies include joint-biased, soft-tissue biased, or needling interventions, whereas top-down strategies include exercise and cognitive interventions. Evidence shows that the effectiveness of these interventions depends on the application of proper clinical reasoning, since not all strategies are effective for all headaches. For instance, evidence of non-pharmacological interventions is more controversial for migraines than for tension-type or cervicogenic headaches, since migraine pathogenesis involves activation of sub-cortical structures and the trigemino- vascular system, whereas pathogenesis of tension-type or cervicogenic headaches is most associated to musculoskeletal impairments of the cervical spine. We conclude that current literature suggests that not all non-pharmacological interventions are effective for all headaches, and that multimodal, not isolated, approaches seem to be more effective for patients with headaches. Most published studies have reported small clinical effects in the short term. This narrative literature review provides some hypotheses for discrepancies in the available literature and future research. Clinical reasoning should be applied to better understand the effects of non-pharmacological interventions.

## 1. Introduction

In the 21st century, headache is probably the most common clinical syndrome most attended by neurologists. Headache causes substantial pain and related-disability to the individuals and it is associated with considerable costs to health care systems [1]. Headaches are classified as primary or secondary headaches. Primary headaches are those that are not the result of an underlying medical specific condition and mainly include migraine, tension-type, and trigeminal autonomic cephalalgias [2]. Secondary headaches are those occurring for the first time in close temporal relation to a specific disorder known to cause headache, e.g., headache attributed to trauma or injury to the head and/or neck [2]. Migraine and tension-type headache (TTH) are the most prevalent primary headaches, whereas cervicogenic headache (CeH) is the most prevalent secondary headache [3,4]. The Global Burden of Disease Study ranked headaches as the second leading cause of years lived with disability worldwide [5].

The International Classification for Headache Disorders (ICDH-III) presents headache diagnosis in a hierarchical fashion with migraine as the first consideration, TTH as the second, and CeH and other headaches next [2]. Nevertheless, current management of any headache involves pharmacological and non-pharmacological interventions. Non-pharmacological interventions are commonly used by people with TTH, migraine, and CeH, manual therapy being the most common non-medical treatment requested by these patients [6]. In fact, non-pharmacological therapies are included on most international guidelines related to the management of TTH and migraine. For instance, the European Federation of Neurological Societies (EFNS) Guidelines suggest that non-pharmacologic management should be considered into the multimodal management of people with TTH, although their evidence is still conflicting [7]. Similarly, the Italian Guideline for primary headache also includes non-pharmacological intervention as complementary treatment for patients with TTH and migraine, particularly in those where the pharmacological treatment may be not a good strategy option, e.g., pregnancy, poor tolerability of drugs, or childhood [8]. Interestingly, non-pharmacological treatment represents self-management strategies commonly used by patients with headaches. Probyn et al. found that self-management interventions were more effective for reducing headache intensity (Standardized mean differences (SMD) −0.36, 95% confidence interval (CI) −0.45 to −0.26) and headache-related disability (SMD −0.32, −0.42 to −0.22) than usual care for TTH and migraine [9].

Scientific evidence of non-pharmacological interventions is highly heterogenous and several systematic reviews have been conducted with contradictory results. For instance, systematic reviews conducted by Chaibi et al. concluded that: (1) the efficacy of physical therapy seems to be similar than the efficacy of tricyclic antidepressants at mid-term in chronic headaches [10]; (2) multimodal approaches including manual therapy and relaxation can be equally effective as propranolol and topiramate in the prophylactic treatment of migraine [11]; and (3) there have been few randomized clinical trials including a control group in CeH [12]. However, the results from more recent meta-analyses have been slightly different. Mesa-Jiménez et al. concluded that multimodal manual therapy interventions were more effective than pharmacological medication for reducing the frequency (SMD −0.80, −1.66 to −0.44), intensity (SMD −0.59, −0.88 to −0.30), and duration (SMD, −0.55, −0.91 to −0.19) of the headache in short-term, but not long-term, TTH [13]. Similarly, Luedtke et al. showed that manual therapy was effective for reducing pain intensity in TTH (MD −1.11, −1.64 to −0.57), CeH (MD −2.52, −3.86 to −1.19) and migraine (MD −1.94, −2.61 to −1.27), and also headache duration in migraine (MD −22.4 h, −33.9 to −10.9) and CeH (MD −1.7 h per day, −2.1 to −1.2) [14]. Finally, a recent meta-analysis has concluded that manual therapies are effective for increasing health-related quality of life (MD −2.47, −3.27 to −1.68) and related-disability (MD −5.62, −10.69 to −0.54) in TTH and migraine, while its effects on CeH were inconsistent [15]. This evidence supports the effectiveness of non-pharmacological manual therapy interventions for the treatment of headache; however, these assumptions should be considered with caution due to the heterogeneity in study designs and manual therapy approaches applied [16].

Further, chronic headaches and non-pharmacological interventions are broad terms including different headaches and different therapeutic approaches. In fact, published systematic reviews and meta-analyses only assessed isolated interventions (e.g., spinal manipulation) on a specific headache types (e.g., migraine or TTH), and results are somewhat conflicting. Since current evidence suggests that non-pharmacological interventions may play an important role in headache treatment, it is important to identify gaps in existing knowledge in relation to the clinical reasoning to be applied for identification of the proper therapeutic strategies to specific headaches. Therefore, we conducted a narrative literature review aimed to: (1) discuss relevant pain mechanisms underlying non-pharmacological interventions; (2) identify and map the most updated scientific evidence for non-pharmacological therapies in TTH, migraine, and CeH; and (3) discuss the clinical reasoning explaining discrepancies on effects of non-pharmacological interventions based on their underlying nociceptive mechanisms.

## 2. Bottom Up (Hands-ON) or Top Down (Hands-OFF) Interventions

Proper management of chronic pain syndromes, including headaches, have begun to target various pain mechanisms as one strategy for combatting chronic pain [17]. In fact, clinical and basic science evidence support that proper management of patients with headache should be multimodal, and consider individualized and personalized patient’s perspectives by including passive and/or active strategies, active listening, empathy, and psycho-social issues. This is particularly important in individuals with headache, since it is helpful to encourage patients to choose among the various treatment options after proper explanation of the benefits and risks of each therapeutic approach. For instance, it is important that headache sufferers are informed about the potential benefits and risks of prolonged use of pharmacological interventions.

Additionally, interventions for people with headache should be based on current knowledge of nociceptive pain mechanisms. There is evidence supporting the presence of an altered nociceptive pain processing both TTH [18] and migraine [19]. Key indicators of central sensitization include presence of widespread hyperalgesia, allodynia, absence of conditioned pain modulation, and sleep disturbances. All these features are present in patients with migraine or TTH, particularly in those with the chronic form.

**Figure 1:**
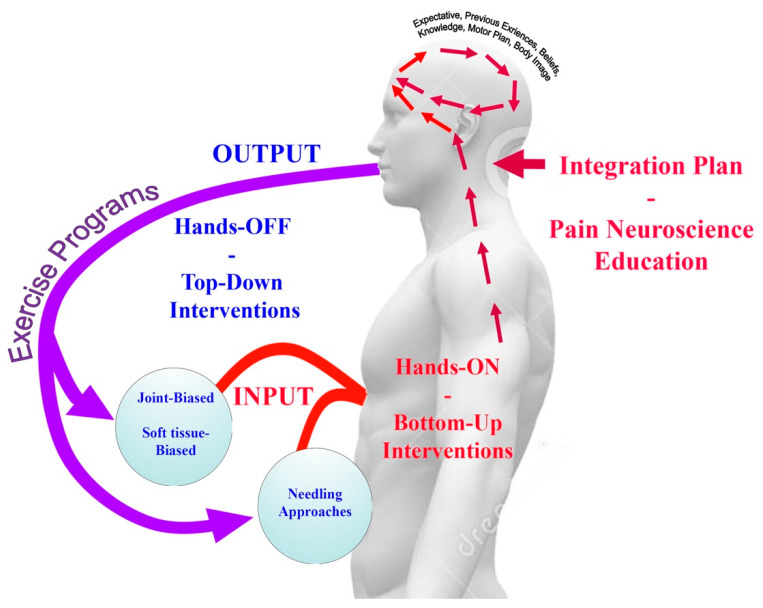
Therapeutic model integrating bottom-up and top-down interventions for the management patients with headache.

Central sensitization is initiated by long-lasting/prolonged peripheral noxious inputs (peripheral sensitization); however, once central sensitization has been established, only minimal nociception is required to maintain the sensitization state [20]. Hence, clinical management of headache patients needs to extend beyond tissue-based impairment (bottom-up interventions) to incorporate treatment strategies directed at normalizing central nervous system sensitization (top-down interventions). Therefore, proper clinical identification of peripheral and central sensitization drivers is important [21]. From a clinical perspective, in those individuals primarily mediated by peripheral mechanisms (bottom-up sensitizers) appropriate and early treatments targeted to those tissues potentially related to the peripheral input, e.g., muscular or articular, and functional activities, e.g., local exercises targeting the specific musculoskeletal disorders associated to the headache, should be encouraged. In those patients primarily mediated by central mechanisms (top-down sensitizers), multimodal treatments including physical, psychological, or cognitive and educational approaches should be applied. This clinical reasoning is, in fact, supported by current understanding of the potential neurophysiologic mechanisms underlying the effects of physical manual therapies [22]. In fact, current trends support the combination of bottom-up (Hands-ON) and top-down (Hands-OFF) therapy interventions in the management of chronic pain (Figure 1) [23].

Current therapeutic models should integrate the use of bottom-up (Hands-ON) and top-down (Hands-OFF) interventions where the peripheral stimuli induced by any bottom-up (Hands-ON) strategy is able to precipitate and modulate different responses at spinal cord, sympathetic nervous system and brainstem [22]. In such a scenario, the context, placebo expectancies, patient’s expectances and previous experiences are integrated into an integration plan in the brainstem. This integration plan is related to potential placebo effect inherent to non-pharmacological interventions, particularly bottom-up (Hands-ON). The effects of placebo should not be ignored in any therapeutic intervention since it is associated with an activation of pre-frontal and anterior cingulate cortex, the thalamus, insula, amygdala, and periaqueductal grey neurons, all pain mechanisms also involved in non-specific effects of manual therapies [22]. In addition, the nature of the treatment, the personal interactions, patient’s expectances and treatment repetition are of importance for the placebo effect. Since most bottom-up (Hands-ON) strategies involve manual contact and patient-therapist personal interaction, the placebo effect should be highly considered [24]. It has been reported that at least 25–35% of the effects of prophylactic treatment for headaches is placebo, although this rate is higher, until 50–60% in children with headache [25]. However, there has been no study investigating the placebo effect of most of non-pharmacological interventions in headache.

## 3. Literature Search

This narrative literature review was based on a methodological framework for scoping reviews described in Preferred Reporting Items for Systematic Reviews and Meta-Analyses Extension for Scoping Reviews (PRISMA-ScR) [26], but slightly modified because of the multidimensional nature of the topic.

### 3.1. Search Strategy

A search strategy based on potential themes and topics considered relevant to the headache field according to the current literature and hot topics was conducted. Electronic literature searches were conducted on the following database from January 2000 to January 2020: MEDLINE, EMBASE, AMED, CINAHL, EBSCO, PEDro, Cochrane Database of Systematic Reviews, PubMed, Cochrane Collaboration Trials Register, and SCOPUS. We also screened the reference lists of the papers that were identified in the database. Database search strategies were conducted with the assistance of an experienced health science librarian.

To avoid missing any relevant study in the search, the following broadly heading terms were combined by using Boolean operators in the search strategy: “tension type headache”, “migraine”, “cervical headache”, “cervicogenic headache”, “manual therapy”, “physical therapy”, “exercise”, “muscle”, “joint”, “spinal manipulation”, “spinal mobilization”, “dry needling”, “soft tissue”, “trigger point”, “neck”, “cervical spine”, “cognitive behavior”, “clinical reasoning”, “psychological treatment”, “pain mechanisms”.

### 3.2. Inclusion Criteria and Data Extraction

We mainly considered systematic reviews, meta-analyses, randomised controlled trials, and expert opinions published in the last 20 years discussing either neurophysiological mechanisms, clinical reasoning or effectiveness of non-pharmacological interventions in TTH, migraine, and CeH. Two authors reviewed the identified publications on relevance by checking and by judging the title and abstract. Authors included the relevant papers on each of the topic based at personal discretion. Discrepancies in reviewers’ responses at any stage of the screening were resolved by asking a third author.

### 3.3. Data Mapping

After the data extraction, we organized the literature thematically, according to the following topics: (1) mapping of theoretical aspects of non-pharmacological interventions; (2) summarizing the most updated scientific literature about effectiveness of non-pharmacological interventions grouped by targeted tissue and headache; or, (3) identify research gaps in the existing literature and propose hypotheses for better understanding of the current literature.

## 4. Scientific Evidence of Joint-biased Interventions for Headaches

The electronic searches identified 265 potential papers related to joint-biased interventions for headaches. After removing duplicates, 100 papers remained. Eighty-four were excluded based on examination of the abstracts, leaving 16 papers for discussion in the narrative review based on their relevance and upload date (*n* = 4 justifying rational of joint-biased interventions; *n* = 9 systematic reviews/meta-analyses discussing most updated evidence, and *n* = 3 for discussing potential adverse events).

The rational for the application of joint-biased interventions is mainly based on the presence of musculoskeletal disorders of the cervical spine in headache sufferers. An international Delphi study found that physiotherapists evaluate several musculoskeletal impairments of the cervical spine related to joint-biased interventions, e.g., manual joint palpation, cervical flexion-rotation test, cervical range of motion, passive physiological intervertebral movements, reproduction/resolution of headache symptoms and combined movement tests [27]. A recent meta-analysis concluded that the cervical flexion-rotation test has a pooled sensitivity of 0.83 (95%CI 0.72–0.94) and specificity of 0.82 (95%CI 0.73–0.91) for the assessment of patients with CeH [28]. In addition, the high prevalence of neck pain in primary headaches, such as TTH and migraine [29], may also benefit from joint-biased interventions. It is, therefore, conceivable that the cervical spine contributes to headache symptoms. and that joint-biased interventions could be effective.

Joint-biased interventions include cervical manipulation and mobilization therapies and have been advocated to be effective for chronic headaches by different professions, e.g., physical therapists, osteopaths, and chiropractors. Joint thrust manipulation consists of high velocity-low amplitude movements delivered at the end, but normal anatomical, range of motion; joint mobilizations consist of low velocity moderate-high amplitude movements (rhythmical) delivered at the end of the available restricted joint motion [30].

Scientific evidence of joint-biased interventions for the management of headache is highly conflicting. Previous systematic reviews, including the early Cochrane Review, concluded that there was insufficient evidence to support or refute the efficacy of spinal manipulation in TTH [31,32], or migraine [33]. On the contrary, Chaibi et al. found that spinal manipulation was equally effective as some medication in the prophylactic management of migraine [11]. Discrepancies can be related to the fact that joint-biased interventions may be not equally effective for different headaches [34]. For instance, systematic reviews analyzing the effects of spinal manipulation in patients with CeH obtained more positive results for this secondary headache [35,36]. The most recent meta-analysis has pointed out that spinal manipulation may be effective for reducing the number of migraine days (Hedges’ g: −0.35, 95%CI −0.53 to −0.16) and the intensity in migraine (Hedges’ g: −0.28, −0.46 to −0.09) [37]. However, the effects were small and only assessed at short-term [37]. Similarly, a more recent review has also concluded that cervical spine manipulative therapy may be effective for CeH patients [38]. However, an important limitation of current evidence about joint-biased clinical trials includes the lack of information on which specific manipulation or mobilization intervention was used, the targeted level or the clinical reasoning for applying any technique, combination of both joint manipulation and mobilization techniques in most studies or combination of joint-biased interventions with other approaches, e.g., soft tissue-biased approaches. Therefore, it is not possible to determine the effects of spinal thrust joint manipulation or non-thrust mobilization interventions for the management of particular headaches.

Additionally, the use of cervical manipulations, mostly related to those targeting the upper cervical spine, remains controversial because of subsequent concerns about their safety. Adverse reactions to manipulative therapy range from minor events, e.g., pain or limitation in range of motion, to more serious events, e.g., vertebral or carotid arteries dissection [39]. Puentedura et al. reported that if all contraindication and red flags are ruled out, there is potential for a clinician to prevent 44.8% of adverse events associated with upper cervical spine manipulations; but clinicians should consider that around 10.5% of minor events are unpreventable [40]. A recent review of reviews concluded that it is not possible to provide an overall conclusion about safety of upper cervical manipulation because contradictory results of published reviews: 46% expressed that cervical spine manipulation was safe, 13% expressed that it is harmful, and 42% that were neutral or unclear [41].

## 5. Scientific Evidence of Soft Tissue-biased Interventions for Headaches

The electronic searches identified 145 potential papers related to soft tissue-biased interventions for headaches. After removing duplicates, 90 papers remained. Eighty (*n* = 80) papers were excluded based on examination of the abstracts, leaving 10 papers for discussion in the narrative review based on their relevance and upload date (*n* = 5 justifying the rational of soft tissue-biased interventions; *n* = 3 systematic reviews/meta-analyses and *n* = 1 randomized clinical trial for discussing most updated evidence, and *n* = 1 expert opinion).

Similarly, than with joint-biased interventions, clinical reasoning supporting the application of soft-tissue interventions for individuals with TTH, migraine or CeH is mainly based on the potential role of muscle tissues in these disorders [42], although this is most accepted for TTH [43,44]. This is supported by clinical experience since trigger point (TrP) examination is one of the musculoskeletal impairments most examined and also treated by physical therapists in headache patients [27].

Soft tissue-biased interventions include a broad number of strategies targeting the muscle and fascia tissues including pressure interventions, massage, stroke, stretching, or myofascial release [45]. All these soft tissue-biased interventions have the aim to decrease muscle tension by providing proper proprioceptive inputs to the central nervous system [45]. The therapist decides which soft tissue intervention is applied based on clinical reasoning and examination of muscle tissues.

A recent meta-analysis has concluded that manual therapies targeting muscular TrPs reduce frequency, intensity, and duration of attacks in TTH (MD −3.5 attacks per month, −4.91 to −2.09; −12.8 on a 100mm visual analogue scale; −19.49 to −6.17; −0.51 h/attack, −0.97 to −0.04) and migraine (MD −1.95 attacks/month, −3.0 to −0.8; −13.6 on a 100mm visual analogue scale, −19.54 to −7.66; −10.68 h/attack, −14.41 to −6.95), although the quality of evidence was still low [46]. Again, most studies investigated short-term effects and no data about the duration of headache relief is available. The role of soft tissues has been also investigated in CeH, but to a lesser extent. A systematic review concluded that manual therapies targeted to either muscle or joints of the upper cervical spine is an effective treatment for CeH, but this conclusion should be considered with caution since studies mostly included patients with infrequent CeH [12]. Castien et al. found that the application of a manual therapy program mainly based on soft-tissue interventions was more effective than general practice for the management of CTTH [47]. However, it seems that multimodal approaches are more effective for reducing pain in TTH [48] and CeH [49].

## 6. Scientific Evidence of Needling Therapies Interventions for Headaches

The electronic searches identified 95 potential papers about needling therapies for headaches. After removing duplicates, 40 papers remained. Thirty-three (*n* = 33) papers were excluded based on examination of the abstracts, leaving 7 papers for discussion in the narrative review based on their relevance and update (*n* = 3 systematic reviews/meta-analyses and *n* = 2 randomized clinical trials for discussing most updated evidence, and *n* = 2 narrative reviews).

Needling therapies, including wet (medical doctor) and dry (physical therapist) interventions, are commonly used for the management of people with headaches [50]. Wet needling (injections) involves the application of pharmacological substances, e.g., local anesthetics or botulinum toxin, whereas dry needling involves the use of filament solid needles and no injected substances. Acupuncture and TrP dry needling are the most commonly applied for the management of chronic pain patients [51].

The Cochrane review about the use of acupuncture for TTH concluded that acupuncture is effective for the management of this primary headache, but clinical trials comparing acupuncture with other therapeutic strategies are clearly needed [52]. Likewise, the Cochrane review stated that available evidence suggests that adding acupuncture to symptomatic drug treatment reduces the frequency of migraine attacks/month (SMD: −0.56; −0.65 to −0.48); and that real acupuncture is also more effective than sham-acupuncture, but with a small effect (SMD −0.18, −0.28 to −0.08) [53].

It is important to note that acupuncture is different than dry needling. The main difference between both approaches is that this dry needling inserts the needle on muscle TrPs, and not in standardized acupuncture points. Although the literature suggests that dry needling may be a useful addition to physical therapy, the only systematic review on this topic remarked that further research is required [54]. After this review, some clinical trials have been published. Sedighi et al. compared superficial (skin-biased) versus deep (muscle-biased) dry needing over the suboccipital and upper trapezius musculature in people with CeH and showed that both needling approaches were similarly effective for reducing headaches, but deep dry needling was more effective for improving function [55]. A recent randomized clinical trial found that real dry needling over active TrPs located in the neck and head musculature was more effective than sham needling for reducing headache pain parameters, e.g., intensity, frequency, and duration of headache, and for improving health-related quality of life in patients with chronic TTH [56]. Nevertheless, the number of sessions, muscles which should be needled, dosage, frequency of sessions, and long-term follow-ups are needed to further conclude a real effect of dry needling in primary headaches.

## 7. Scientific Evidence of Exercise Interventions for Headaches

The electronic searches identified 452 potential papers related to exercise and headaches. After removing duplicates, 200 papers remained. One hundred and eighty (*n* = 180) were excluded based on examination of the abstracts, leaving 20 papers for discussion in the narrative review based on their relevance and upload date (*n* = 12 justifying rational of exercises; *n* = 4 systematic reviews or meta- analysis, and *n* = 4 randomized clinical trials discussing most updated evidence).

Since exercise-induced hypoalgesia is mainly related to activation of descending inhibitory pain pathways [57], exercise is of the main top-down intervention used for the management of chronic pain [58]. The inclusion of exercise as a therapeutic approach for headaches has been previously proposed as a strategy for normalization of the central nervous system [59]. However, as different pathogenic mechanisms are involved on each headache, the most appropriate therapeutic exercise program might differ for each one [60].

It is important to consider that there are two different exercise strategies; (1), aerobic exercises; and (2), local exercises targeting specific musculoskeletal disorders of the cervical spine. This differentiation is clinically relevant since aerobic exercises could be the best option in migraine prophylaxis, whereas specific neck/shoulder strengthening exercises may be a potential better choice for TTH [61]. This assumption is based on the presence of motor output changes of the cervical musculature, e.g., reduced neck muscle strength [62], an increased co-activation of the superficial cervical muscles [63], in patients with TTH. In fact, the application of strengthening exercises, ergonomic/posture correction programs and motor control exercise programs have demonstrated to be effective in TTH [64,65,66]. Interestingly, Castien et al. found that neck flexor musculature endurance partially mediates the potential effects of manual therapy interventions in TTH [67]. Nevertheless, it should be noted that there are several studies also showing the presence of similar musculoskeletal disorders of the cervical musculature, e.g., decreased neck muscle endurance [68], increased activity of the superficial muscles during low-load isometric tasks [69], and altered muscle performance [70] in people suffering from migraine. No clinical trial has investigated the effect of specific exercise programs targeting those musculoskeletal impairments of the cervical muscles in individuals with migraine. Finally, there is also moderate evidence supporting neck strengthening and endurance exercise programs for improving pain and function in patients with CeH, although further studies are needed [71].

There is evidence supporting the effectiveness of aerobic exercise for the management of TTH and migraine [72]. A recent meta-analysis found significant reductions in the number of migraine days (MD −0.61 attacks/month, −1.14 to −0.09) after aerobic exercise treatment [73]. However, since migraine pain can worsen with physical activity efforts, the role of exercise in people with migraine is controversial. Nevertheless, although no consensus exists about exercise and migraine, it is well accepted that aerobic exercise may be an option for the prophylactic treatment of migraine in individuals who do not clearly benefit from or do not want to intake drug medication [74]. Considering the minimal side effects of therapeutic exercise, patients with migraine should be encouraged to practice physical exercise; but with proper intensity, frequency, and duration, to achieve the most beneficial clinical outcomes. In fact, a recent meta-analysis concluded that data on the best type of exercise, intensity, and dosage for the management of headaches are still lacking [75]. Another topic for determining relevance is adherence and patient’ satisfaction. Interestingly, adherence to lifestyle habit modifications, including proper and regular practice of exercise, predicted the effectiveness of a multidisciplinary therapeutic program in TTH and migraine [76]. These are important topics since clinicians should take into account that while exercise is able to activate descending inhibitory pain mechanisms, these mechanisms could be slightly different in people with central sensitization, and the opposite outcomes may occur, e.g., unappropriated exercise may induce hyperalgesia if not properly controlled. In such scenario, aggressive or excessive exercise may be detrimental if the intervention triggers sensitized peripheral nociceptors and may cause prolonged pain. This situation plays an important role since kinesiophobia (fear to movement) is present in 50% of migraineurs and is associated to worse cutaneous allodynia severity [77]. Therefore, an inappropriate exercise program could lead to a worse treatment scenario for a headache patient.

## 8. Scientific Evidence of Cognitive Interventions for Headaches

The electronic searches identified 341 potential papers of cognitive/psychological interventions for headaches. After removing duplicates, 185 papers remained. One hundred and sixty-eight were excluded based on examination of the abstracts, leaving 17 papers for discussion in the narrative review based on their relevance and upload date (*n* = 7 justifying rational of cognitive techniques; *n* = 7 systematic reviews or meta- analysis, and *n* = 3 clinical practice guidelines).

Headache is a chronic pain condition also involving psychological aspects of the painful experience [78] where emotional stress is one of the main trigger factors [79]. There is evidence that suggests that headache sufferers exhibit higher anxiety and depression [80,81], and also sleep disturbances [82]. Furthermore, some primary headaches, e.g., migraine, are associated to specific personality traits, e.g., neuroticism [83]. These findings represent the rational basis for psychological management of headaches, particularly considering the complex relationship between mood disorders, sleep quality, burden, and hyperalgesic responses observed in patients with headache [84].

There is substantial evidence in favor of psychological treatments for headaches; but further research on which specific intervention (cognitive-behavior therapy, coping strategies, relaxation training, biofeedback, mindfulness-based treatment, or autogenic treatment) is also needed [85]. A recent meta-analysis concluded that psychological treatments were effective for reducing the frequency of headache (SMD −0.7, −1.22 to −0.18) with no differences between migraine or TTH, or depending on psychological intervention [86]. Similarly, the Cochrane Review also found that psychological treatments were effective for reducing pain in children and adolescents with headache, but no effect on depression or anxiety was observed [87]. It is also important to note that there are some psychological interventions, e.g., behavioral and cognitive strategies, targeting specific aspects of the emotional spectrum, e.g., sleep disorders. The meta-analysis conducted by Sullivan et al. has concluded that cognitive behavior therapy for sleep disorders was effective for improving headache frequency and sleep quality in patients with headache [88].

Different meta-analyses have also concluded that biofeedback is effective for reducing the intensity and the frequency of headache attacks in TTH [89] and migraine [90]. Based on these results, the use of biofeedback is recommended in some European guidelines, e.g., EFNS or Italian, for the management of TTH and migraine [7,8], but not in others, e.g., the National Institute for Health and Care Excellence (NICE), United Kingdom [91]. Discrepancies between guideline recommendations are based on the lack of high-quality evidence supporting biofeedback use. It could be hypothesized that, since the objective of biofeedback is to teach the patients to manage their muscle tension in those activities that they associate with their headaches, biofeedback would be mostly effective in individuals with headaches associated to pericranial tenderness.

Of particular interest could be the use of therapeutic pain neuroscience education. Therapeutic pain neuroscience education can be defined as educational sessions, usually directed by health care professionals, providing patients’ knowledge about underlying mechanisms of their condition and proper skills to manage their lives. A meta-analysis reported strong-moderate evidence for intermediate effects of therapeutic neuroscience education on headache frequency (SMD −0.24, −0.48 to −0.01) and related-disability (SMD −1.02, −1.95 to −0.08) in people with migraine [92]. No effects on self-efficacy or depressive symptoms were found.

Finally, the problem with cognitive/psychological treatments in clinical setting is that they are more time consuming than, e.g., pharmacological treatment, and require the active participation and motivation of the individual. Further, treatment costs and their coverture by health care systems largely differs in some countries.

## 9. Summarizing the Literature: which Headache Patients Can Benefit from Non-Pharmacological Interventions?

This literature review has summarized the most updated current evidence and hypotheses about non-pharmacological interventions for the treatment of TTH, migraine or CeH. It seems that most non-pharmacological interventions have a small clinical effect on these headaches, and most importantly is that not all treatment strategies are indicated for all headache syndromes (Table 1).

It is important to consider that not all headache patients will benefit from non-pharmacological interventions. It is probably that patients with more severe headaches attending to specialized clinics of chronic pain will seek more frequently for this kind of treatment. Nevertheless, current literature also supports that non-pharmacological interventions are also effective for patients with the episodic forms of TTH or migraine, patients which are more frequently attended at primary care.

There is preliminary evidence trying to identify subgroups of patients who could be most likely to benefit from some specific non-pharmacological interventions, particularly soft tissue-biased strategies. In fact, it has been suggested that not all patients with headache will benefit from manual therapy [93]. In line with this hypothesis, some studies have tried to identify prognostic variables to guide non-pharmacological interventions in patients with TTH or CeH. For instance, Jull et al. found that the absence of migraineurs features such as photophobia was associated with better outcomes after the application of spinal manipulation/mobilization and exercise therapy in patients with CeH [94]. Fernández-de-las Peñas et al. reported that patients with chronic TTH exhibiting more cervical musculoskeletal impairments experienced better short-term outcomes after the application of joint mobilization and soft tissue techniques [95]. The study by Castien et al. found that higher headache intensity, absence of multiple-site pain, and cervical muscle impairment were factors associated with better outcomes in patients with TTH [96]. Current evidence would suggest that patients with lower degree of central sensitization but with musculoskeletal impairments in the neck will benefit to a greater extent from non-pharmacological manual interventions, although further trials are needed.

## 10. Clinical Reasoning for the Management of Headaches

The existence of a wide range of management interventions commonly applied for headaches without clear clinical effectiveness of their efficacy can be the main consequence of an inconclusive understanding of the underlying pathophysiology or an inappropriate clinical reasoning considering these headaches (TTH, migraine, CeH) equally [59]. The challenge facing clinicians treating patients with headaches with non-pharmacological strategies is how to select the most appropriate treatment approach for each particular individual, who is likely to be different in his/her clinical presentation [92]. For choosing the appropriate therapeutic intervention, clinicians must interpret and identify the predominant underlying pain mechanisms in a particular headache patient [59].

In fact, effectiveness of physical therapy is different on each headache disorder; for instance, evidence in migraine is more controversial than in TTH. One potential explanation could be the fact that migraine pathogenesis involves the activation of sub-cortical structures as well as the trigemino-vascular system, whereas the pathogenesis of TTH is essentially considered a generation of muscle/myofascial pain. Similarly, it is expected that spinal manipulation/mobilization would be effective for CeH because this secondary headache is considered a referred pain elicited by nociceptive stimulation of upper cervical joints (C1-C3). Accordingly, individuals with painful disorders of upper cervical zygapophyseal joints may exhibit significant benefits with treatments (joint-biased) directed at cervical pain generators [97]. On the contrary, since the pathogenesis of TTH is based on myofascial pain disorders, it is expected that spinal joint manipulation would be not as effective as in CeH.

Another clinical reasoning would be that headache patients presenting with musculoskeletal impairments of the neck may particularly benefit of non-pharmacologic bottom-up approaches [98]. A recent meta-analysis found that musculoskeletal pain disorders of the cervical spine seem to be more present in individuals with TTH than in those with migraine [99], but this assumption should be considered with caution since migraine patients also exhibit musculoskeletal disorders of the cervical spine when compared to healthy controls [100]. In fact, the role of the cervical spine in headaches, and, therefore, CeH was not accepted as a valid diagnosis, until the third edition of the ICHD [2], and the differentiation from migraine-related neck pain, TTH with associated neck pain or CeH is still a very “blurred” line of distinction. Interestingly, this conclusion is the same of a recent clinical practice guideline recommending that clinicians managing patients with headache should rule out migraine as the cause of headaches and classify headaches associated with neck pain as TTH or CeH once other sources of headache pathology has been ruled out [101].

On the contrary, in those patients with co-morbid anxiety and mood disorders, problems for managing stress, significant headache-related disability and/or medication overuse, cognitive strategies and exercise programs should be better applied. Therefore, it is clinically important to identify, if possible, the main sensitizer driver on a particular patient. Nevertheless, clinical and scientific evidence supports the conclusion that proper management of patients with headaches should be multimodal, including appropriate use of pharmacological (e.g., acute/abortive and preventive drugs) and non-pharmacological (e.g., manual therapies, exercises, biofeedback, and needling therapies) interventions.

## 11. Future Research Questions

The current literature review has identified several gaps that need to be addressed in future studies. First, most evidence has mainly investigated short-term effects, so we do not currently know the duration of pain relief. This is a relevant topic since headaches are chronic pain syndromes of long duration. Our literature review proposes some hypotheses behind a potential clinical reasoning explaining the conflicting results observed in the current literature in relation to the effectiveness of non-pharmacological interventions. Future studies should introduce the propose therapeutic model based on a clinical reasoning considering nociceptive pain mechanisms for improving the clinical outcomes in specific subgroups of patients with TTH, migraine or CeH.

Another important topic for investigating in future studies would be cost-effectiveness of these non-pharmacological interventions. This is a relevant topic to investigate since non-pharmacological therapies are usually time and personnel consuming and also relatively expensive which could limit their availability to specialty centers and public health care system in many cases. In fact, the is a lack of studies in this area. Only two studies have investigated the costs associated to the use of acupuncture for the management of headache and both suggest a potential beneficial cost for this intervention, although future studies are needed [102,103].

## 12. Conclusions

This literature review summarizes most updated data on non-pharmacological treatments for patients with TTH, migraine, or CeH and the underlying clinical reasoning explaining discrepancies their therapeutic effects. Several non-pharmacological interventions have shown positive effects for the management of these headaches, but their clinical relevance was small and the duration of the effect was mainly in the short-term. It is important to identify, if possible, the main sensitizer driver for proper clinical reasoning about the selection of the appropriate non-pharmacological therapeutic strategy. In addition, not all patients will benefit from the same interventions and identification of these subgroups of patients should give future research. Appropriate therapeutic management of patients with headache should be multimodal, including beyond tissue-based impairment therapies (bottom-up interventions) and central nervous system interventions (top down interventions).

### Key Findings

1. Treatment strategies used for the management of headache should include beyond tissue- based impairment therapies (bottom-up interventions) and central nervous system interventions (top down interventions).
2. Bottom-up (hand-on) strategies can include joint-biased, soft-tissue biased, and needling therapies whereas top-down (hand-off) strategies include exercise and cognitive interventions.
3. Evidence shows that the effectiveness of non-pharmacological interventions for headaches depends on a clinical reasoning since not all strategies are equally effective for all headaches.
4. Evidence of non-pharmacological interventions is more controversial for migraine than for TTH or CeH, since migraine pathogenesis mainly involves activation of sub-cortical structures and the trigemino-vascular system whereas pathogenesis of TTH or CeH is associated to musculoskeletal impairments of the cervical spine.

## Figures and Tables

**Table 1:** Effects of non-pharmacological interventions in tension-type headache (TTH), migraine and cervicogenic headache (CeH).

Intervention	Tension Type Headache	Migraine	Cervicogenic Headache
Joint-biased interventions	−	+/−	+
Soft-tissues interventions	+	+/−	?
Needling interventions			
Acupuncture	+	+	?
Dry Needling	?	?	?
Exercises			
Localized	+	?	+
Aerobic	+	+	?
Cognitive Interventions	+	+	?
Pain Neuroscience Education	?	+	?
